# High-Throughput Sequencing Reveals a Dynamic Bacterial Linkage between the Captive White Rhinoceros and Its Environment

**DOI:** 10.1128/spectrum.00921-23

**Published:** 2023-07-06

**Authors:** Xiaojun Zhong, Junyang Zhao, Ying Chen, Yanxin Liao, Tao Qin, Dingjiang Zhang, Xiaogang Lai, Chunlong Yang, Yu Wang, Xianfu Zhang, Menghua Yang

**Affiliations:** a College of Animal Science and Technology, College of Veterinary Medicine, Zhejiang A&F University, Key Laboratory of Applied Technology on Green-Eco-Healthy Animal Husbandry of Zhejiang Province, Zhejiang Provincial Engineering Laboratory for Animal Health Inspection & Internet Technology, Hangzhou, China; b Yunnan Shilin Longhui Wildlife Research Center Co., Ltd., Kunming, China; c State Key Laboratory of Subtropical Silviculture, College of Forestry and Biotechnology, Zhejiang A&F University, Hangzhou, China; Texas A&M University

**Keywords:** white rhinoceros, environment, interaction, bacterial community, skin, 16S rRNA sequencing

## Abstract

Soil is an essential part of the animal habitat and has a large diversity of microbiota, while the animal body was colonized by a complex bacterial community; so far, the relationship between the animal host microbial community and the soil microbial ecosystem remains largely unknown. In this study, 15 white rhinoceros from three different captive grounds were selected and the bacterial community of the gut, skin, and environment of these rhinoceros were analyzed by 16S rRNA sequencing technology. Our results showed that *Firmicutes* and *Bacteroidota* were the predominant phyla in the gut microbiome, whereas skin and environment samples share similar microbiome profiles and are dominated by the phyla of *Actinobacteriota*, *Chloroflexi*, and *Proteobacteria*. Although the bacterial composition of the gut differs from that of the skin and environment, the Venn diagrams showed that there were 22 phyla and 186 genera shared by all the gut, skin, and environmental microbes in white rhinoceroses. Further cooccurrence network analysis indicated a bacterial linkage based on a complex interaction was established by the bacterial communities from the three different niches. In addition, beta diversity and bacterial composition analysis showed that both the captive ground and host ages induced shifts in the microbial composition of white rhinoceroses, which suggested that the bacterial linkage between the captive white rhinoceros and its environment is dynamic. Overall, our data contribute to a better understanding of the bacterial community of the captive white rhinoceros, especially for the relationship between the environment and animal bacterial communities.

**IMPORTANCE** The white rhinoceros is one of the world’s most endangered mammals. The microbial population plays a key role in animal health and welfare; however, studies regarding the microbial communities of the white rhinoceros are relatively limited. As the white rhinoceros has a common behavior of mud baths and thus is in direct contact with the environment, a relationship between the animal microbial community and the soil microbial ecosystem appears possible, but it remains unclear. Here, we described the characteristics and interaction of bacterial communities of the white rhinoceros in three different niches, including gut, skin, and environment. We also analyzed the effects of captive ground and age on the composition of the bacterial community. Our findings highlighted the relationship among the three niches and may have important implications for the conservation and management of this threatened species.

## INTRODUCTION

The rhinoceros is one of the world’s most endangered mammals that contains five different species, and the white rhinoceros is the largest of the five species of rhinoceros, which is an herbivore that consumes leaves and stems of monocots or grasses (1–3). Although white rhinoceros have fared slightly better than other rhinoceros, the numbers of white rhinoceros in the wild have shown a dramatic decrease associated with the ongoing poaching crisis and habitat loss (1–3). Many studies have demonstrated that the gut microbiome is essential for many biological processes within their hosts and participated in host health, including metabolism, intestinal homeostasis, immune maturation, and regulation (4–7). Dysbiosis or disruption of the host gut microbiome was associated with multiple immunological, metabolic, and developmental disorders (8, 9). Given the significant impact of the gut microbiota on animal health, it is necessary to figure out the gut microbial structure and function of the white rhinoceros; however, little is known about the microorganisms in this threatened species.

Captive breeding is one of the essential measures for threatened species conservation, and captive ground provides an animal with space for living, recreation, and food production, which is an essential part of the animal habitat. Captive ground, especially soil, is the extensive natural microbial gene reservoir, which has a large diversity of microbiota; and the number of bacterial species in the soil is approximately a factor of 10 higher than that of human feces (10, 11). Most animals’ intestine develops from an initial sterile environment (12, 13), and the composition and structure of the gut microbial community have been shown to be affected by a number of factors, such as host genetics, age, diet, disease, and other environmental factors (14, 15). Previous studies showed that environments with rich microbial diversity could protect against allergies and autoimmune disorders in humans, and the outdoor-associated natural beneficial microbiota indirectly influences the gut microbiome (16–18). As animals are in direct contact with the environment/soil in which they live and drink the water that has passed through soil, a relationship between the gut microbial community and the soil microbial ecosystem appears possible and may closely relate to the host health; however, it remains largely unknown.

Skin is the largest organ of the body, which serves as a defensive system and hosts a wide range of microbiomes (19). The skin-associated microbes participate in the host defensive obstruction against microbial assault (20). Previous studies showed that the homeostasis and physiology of the skin are influenced by gut microbiome (21); however, the relationship between the microbial community of the skin and gut is unclear in many vertebrate species. As both the skin and gut are exposed to the outside environment, an understanding of how the microbes of the skin, gut, and environment interact with each other is also needed and may help improve animal health in captivity.

In this study, 15 white rhinoceros from three different captive grounds were selected and the bacterial community of the gut, skin, and environment of these white rhinoceros were analyzed by 16S rRNA sequencing technology. We attempted to determine the structures and interactions of these bacterial communities of the captive white rhinoceros. In addition, how the diversity of the gut and skin bacterial community changes with captive ground and age was also elucidated here. These findings contribute to a better understanding of the bacterial community of the captive white rhinoceros and have important implications for the conservation and management of this threatened species.

## RESULTS

### Taxonomic characterization and functional predictions of the gut bacterial communities of the captive white rhinoceroses.

**FIG 1:**
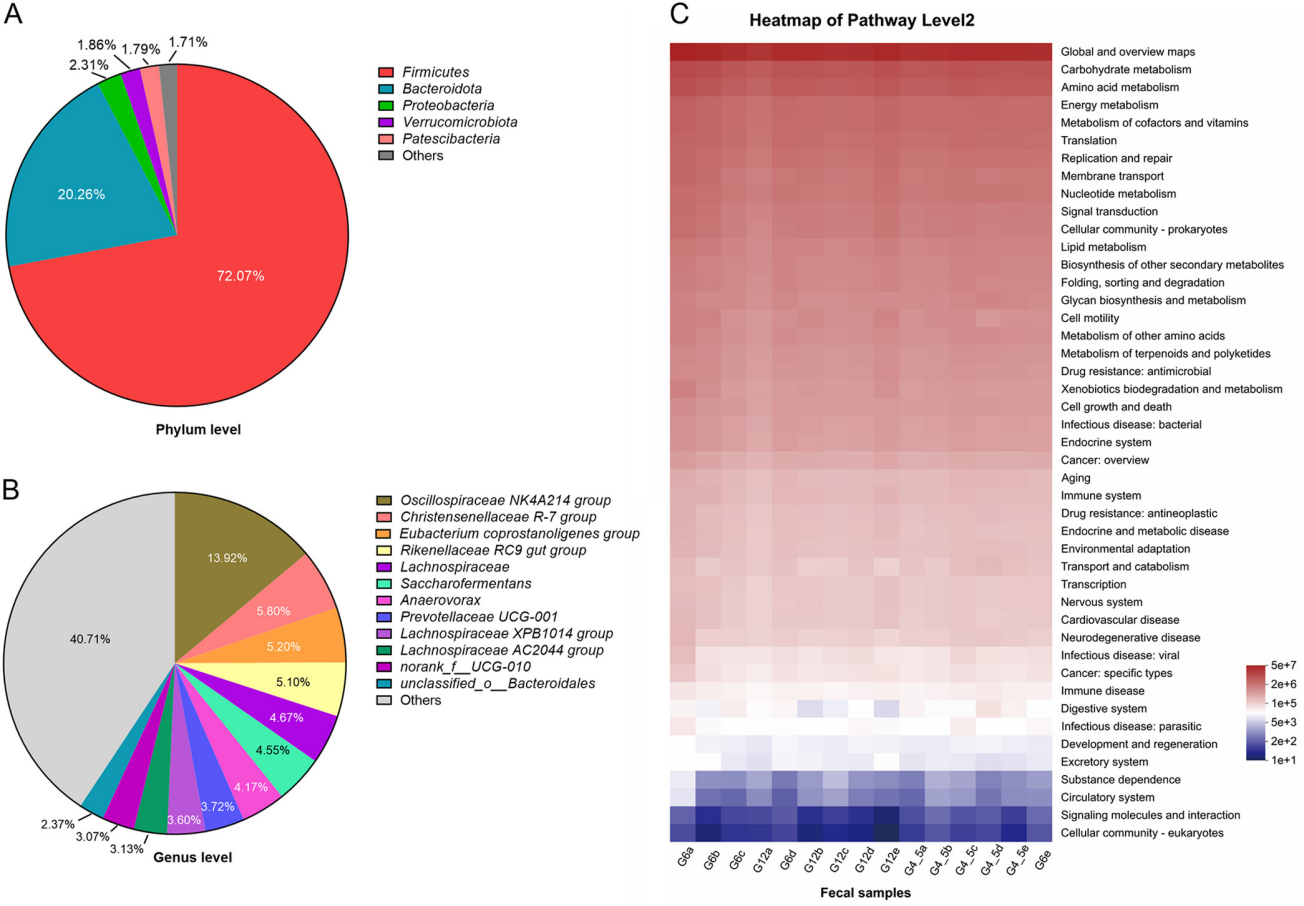
Gut bacterial communities of the captive white rhinoceroses. (A) The relative abundances of gut bacterial community at the phylum level. Phyla with relative abundance < 1% were defined as others. (B) The relative abundances of gut bacterial community at the genus level. Genera with relative abundance < 2% were defined as others. (C) The heatmap shows the relative abundance of bacterial functional categories related to KEGG pathways at level 2. The bacterial functional categories were analyzed by PICRUSt2 based on qualified sequences.

After sequencing, merging, and quality filtering, 600,473 reads (mean of 40,031 reads/sample, *n *= 15 samples) were obtained for the gut bacteria data set of the captive white rhinoceroses. The rarefaction curves generated by Mothur plotting the number of reads by the number of OTUs tended to approach the saturation plateau (Fig. S1). A total of 1,675 OTUs were identified and those classified taxa were assigned to 24 phyla, 50 classes, 102 orders, 171 families, and 327 genera. At the phylum level, the majority of OTUs belonged to the *Firmicutes* (mean per sample = 72.07%), followed by *Bacteroidota* (20.26%), *Proteobacteria* (2.31%), *Verrucomicrobiota* (1.86%), and *Patescibacteria* (1.79%) (Fig. 1A). At the genus level, *Oscillospiraceae* NK4A214 group (13.92%), *Christensenellaceae* R-7 group (5.80%), Eubacterium coprostanoligenes group (5.20%), and *Rikenellaceae* RC9 gut group (5.10%) were the 4 genera above 5% abundance (Fig. 1B). The distribution of functional gene abundance in gut bacteria of the white rhinoceroses was predicted using PICRUSt2 at the second functional level. As shown in Fig. 1C, we found that the top 10 enrichment pathways include global and overview maps, carbohydrate metabolism, amino acid metabolism, energy metabolism, metabolism of cofactors and vitamins, translation, replication and repair, membrane transport, nucleotide metabolism, and signal transduction. As bacterial interaction is important for the stability of healthy biological communities, the cooccurrence network of the gut bacterial communities of the white rhinoceroses was established in this study. We found that the gut bacterial communities of the white rhinoceroses had a complex network module structure, and the number of nodes and edges of *Firmicutes* was obviously higher than the other phylum (Fig. S2).

### The skin and environment of white rhinoceros share similar microbiome profiles.

A total of 572,723 reads (mean of 38,181 reads/sample, *n *= 15 samples) were obtained for the skin bacteria data set, while 543,249 reads (mean of 36,216 reads/sample, *n *= 15 samples) were obtained for the environmental bacteria data set. The rarefaction curves tended to approach the saturation plateau (Fig. S1). In the skin bacteria data set, a total of 5,565 OTUs were identified at 97% sequence identity, with 51 phyla, 147 classes, 323 orders, 527 families, and 1101 genera identified. In the environmental bacteria data set, a total of 5,806 OTUs were identified at 97% sequence identity, with 48 phyla, 150 classes, 334 orders, 549 families, and 1114 genera identified.

**FIG 2:**
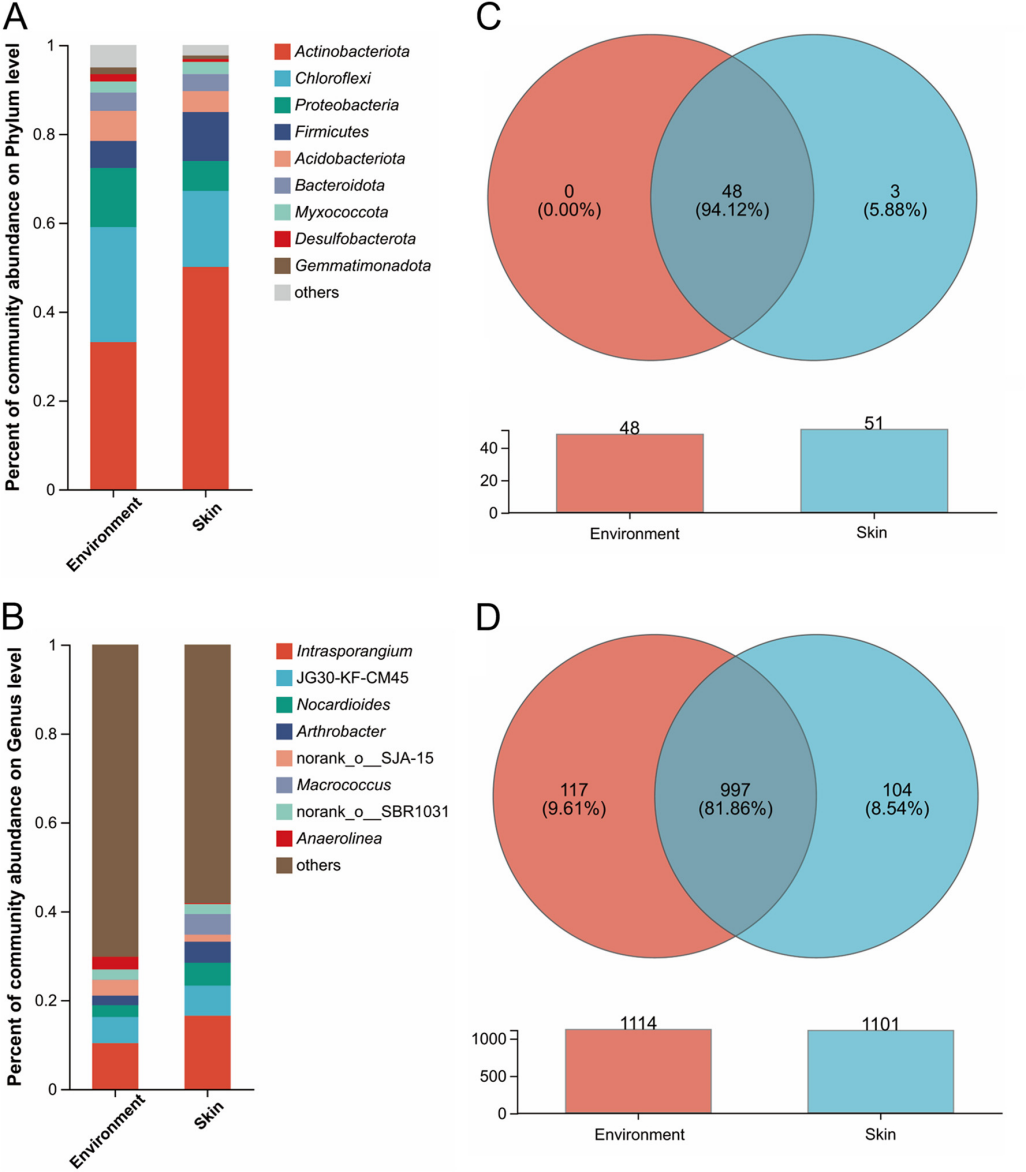
Bacterial composition of the skin and environment of the white rhinoceroses. (A) The relative abundances of the skin and environment bacterial community at the phylum level. Phyla with relative abundance < 1% were defined as others. (B) The relative abundances of the skin and environment bacterial community at the genus level. Genera with relative abundance < 2% were defined as others. (C) Venn diagram based on the phylum level of bacterial communities in the skin and environment of white rhinoceroses. (D) Venn diagram based on the genus level of bacterial communities in the skin and environment of white rhinoceroses.

We found that major phyla and genera of the skin and environmental bacteria data set were similar. As shown in Fig. 2A, the major phyla of *Actinobacteriota* (50% for the skin data set, and 33% for the environmental data set, similarly hereinafter), *Chloroflexi* (17%, and 26%), *Proteobacteria* (6.7%, and 13%), *Firmicutes* (11%, and 6.1%), *Acidobacteriota* (4.7%, and 6.8%), and *Bacteroidota* (3.8%, and 4.1%) contributed to the total microbiome abundance in both the skin and environmental data set. At the genus level, *Intrasporangium* (16% for the skin data set, and 10% for the environmental data set, similarly hereinafter), JG30-KF-CM45 (6.8%, and 5.9%), *Nocardioides* (5.2%, and 2.7%), and *Arthrobacter* (4.7%, and 2.2%) were most abundant genera in both the two bacteria data sets (Fig. 2B). In addition, the Venn diagrams showed that there were large overlaps in both the phyla (94.12%) and genera (81.86%) of the skin and environmental bacteria data set (Fig. 2C and D). We then characterized the beta diversity of the two data sets, and the results showed that the samples from the skin and environment had aggregation (Fig. S3). Taken together, these results indicated that the composition of bacterial communities of white rhinoceroses’ skin and their environment is similar.

### Bacterial composition of the gut differs from the skin and environment in white rhinoceroses.

**FIG 3:**
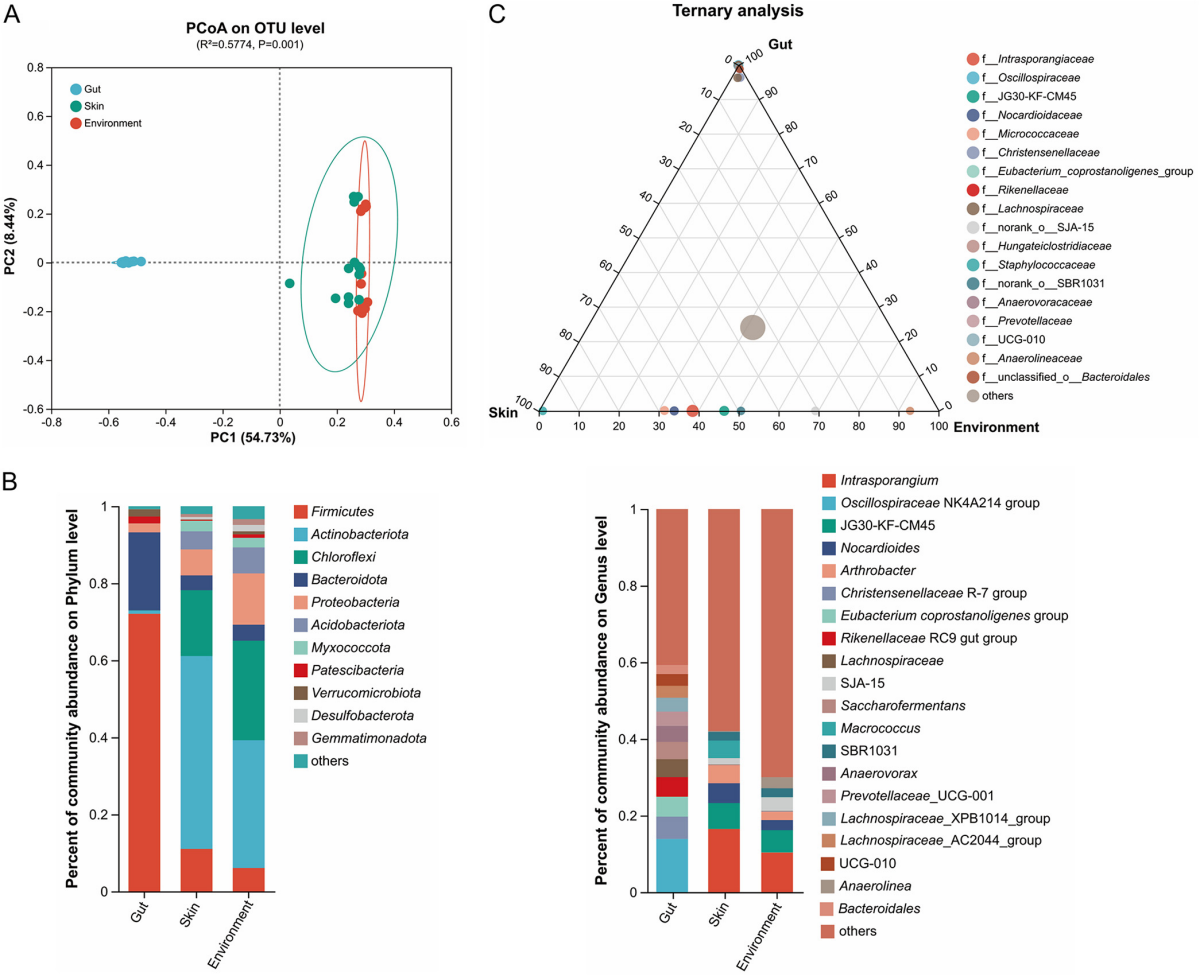
Differentially abundant microbial taxa among gut, skin, and environment of white rhinoceroses. (A) Beta diversity of gut, skin, and environment microbiota displayed in a PCoA scatterplot. The PCoA analysis was performed based on Unweight_UniFrac distance at the OTU level. (B) Relative abundance of dominant microorganisms at the phylum and genus levels. The colors of each block represent different bacterial species, and the height of the block is proportional to the relative abundance. (C) The ternary plot represents the bacterial communities with significant differences in relative abundance at the genus level in the gut, skin, and environment of the white rhinoceroses. The colors of each point represent different bacterial species, and the size corresponds to the relative abundance of each species.

The differences in species abundance distribution among the gut, skin, and environment samples were compared through quantitative analysis of distance in statistics. The beta diversity analysis showed that the samples from the skin and environment had a significant aggregation, whereas the samples of gut were separated from them (Fig. 3A). We then examined potential differences in bacterial taxa among the gut, skin, and environment bacteria data sets at the phylum and genus levels. At the phylum level, the relative abundance of *Firmicutes*, *Bacteroidota*, and *Patescibacteria* was higher in gut data set compared to that in skin and environment data sets, while *Actinobacteriota*, *Chloroflexi*, *Proteobacteria*, and *Acidobacteriota* were lower in gut data set (Fig. 3B). At the genus level, *Oscillospiraceae* NK4A214 group, *Christensenellaceae* R-7 group, Eubacterium coprostanoligenes group, *Rikenellaceae* RC9 gut group, *Lachnospiraceae*, *Saccharofermentans*, *Anaerovorax*, and *Prevotellaceae* UCG-001 were enriched in the gut data set, whereas *Intrasporangium*, JG30-KF-CM45, *Nocardioides*, *Arthrobacter*, and SBR1031 were abundant in the other data sets (Fig. 3B). Moreover, ternary analysis was performed to identify the specific enriched and depleted bacterial genera in different data sets. The results showed that the gut data set was significantly enriched with *Oscillospiraceae*, *Christensenellaceae*, Eubacterium coprostanoligenes group, *Rikenellaceae*, *Lachnospiraceae*, *Hungateiclostridiaceae*, *Anaerovoracaceae*, and *Bacteroidales*, while *Intrasporangiaceae*, JG30-KF-CM45, *Nocardioidaceae*, *Micrococcaceae*, SJA-15, and SBR1031 were significantly depleted (Fig. 3C). These results indicated that the bacterial composition of the gut differs from that of the skin and environment in white rhinoceroses.

### The interaction among the bacterial communities from different niches.

**FIG 4:**
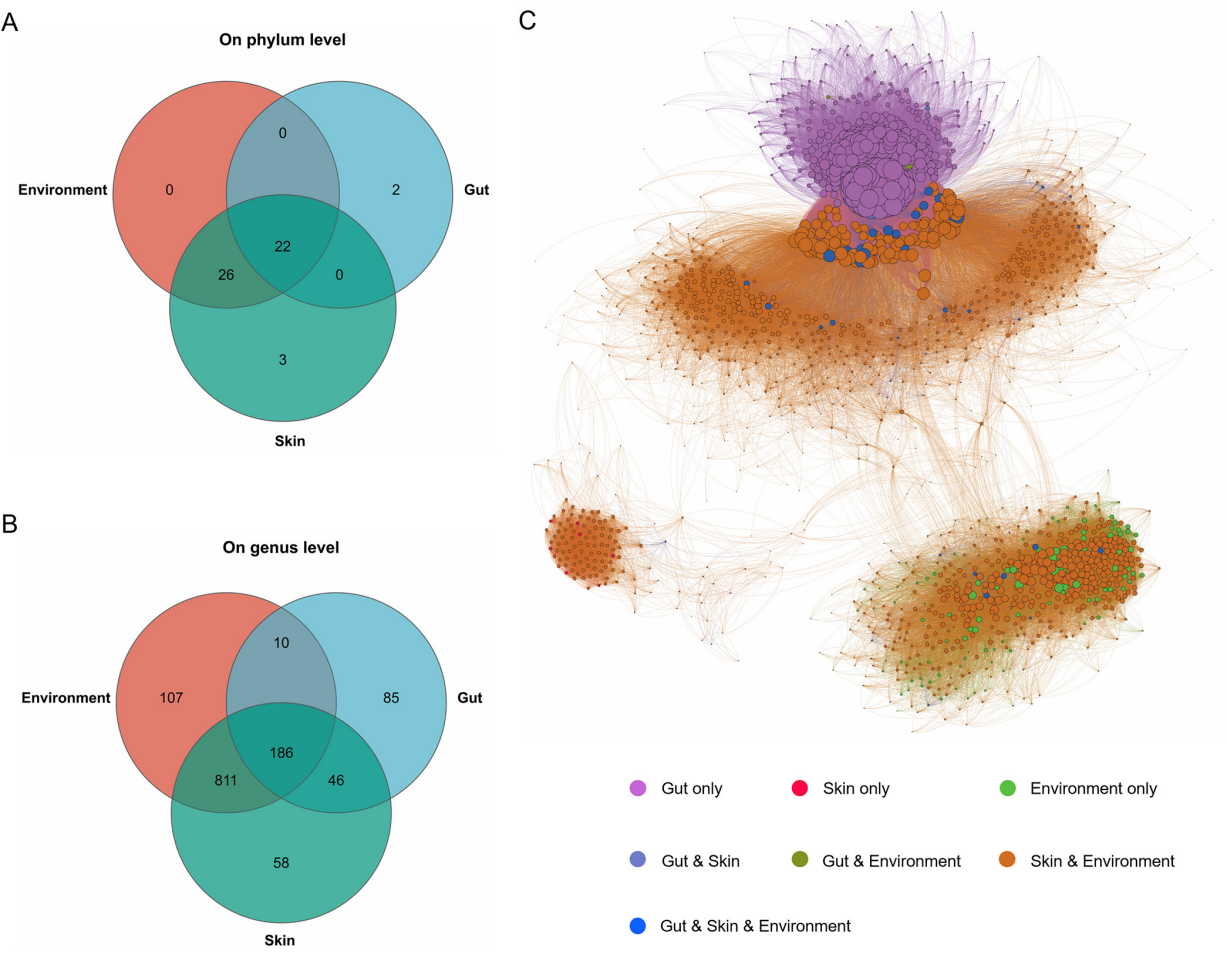
The relationship among the bacterial communities from different niches. (A) Venn diagram based on the phylum level of bacterial communities in the gut, skin, and environment of white rhinoceroses. (B) Venn diagram based on the genus level of bacterial communities in the gut, skin, and environment of the white rhinoceroses. (C) Cooccurrence networks of the microbial communities from the gut, skin, and environment of the white rhinoceroses. The connections in the network represent a strong (Spearman’s *R* > 0.8) and significant (FDR-adjusted *P* < 0.05) correlation. Each node represents a different bacterial species, and the nodes are colored according to their niche assignment. The size of each node represents the relative abundance of the bacterial species.

The Venn diagrams shown in Fig. 4A and B indicate that there were 22 phyla and 186 genera shared by the gut, skin, and environmental bacteria data sets. The overlapped phyla mainly included *Firmicutes* (30.93%), *Actinobacteriota* (27.60%), *Chloroflexi* (13.95%), *Bacteroidota* (9.71%), and *Proteobacteria* (7.30%), while the overlapped genera mainly included *Intrasporangium* (14.01%), *Oscillospiraceae* NK4A214_group (7.78%), JG30-KF-CM45 (6.58%), *Nocardioides* (4.10%), and *Arthrobacter* (3.62%) (Fig. S4A and B). At the genus level, we also found that the overlaps (46 genera) between gut and skin data sets were larger than the overlaps (10 genera) between gut and environment data sets. Thereinto, the overlaps of gut and skin data sets mainly included *Prevotellaceae* UCG-001 (26.65%), *Lachnospiraceae* AC2044 group (22.42%), *Prevotellaceae* (10.97%), and UCG-005 (9.06%) (Fig. S4C); in contrast, the overlaps of gut and environment data sets mainly included *Hydrogenoanaerobacterium* (41.08%), *Mogibacterium* (26.45%), *Erysipelotrichaceae* (12.24%), *Peptostreptococcales Tissierellales* (7.77%), and *Lachnospiraceae* UCG-010 (6.62%) (Fig. S4D). A cooccurrence network pattern was further applied to investigate the interactions among the bacterial communities from the three niches. As shown in Fig. 4C, we observed high clustering coefficients of bacterial species of the gut, skin, and environment, while the individual clustering regions also existed. These results indicated that bacterial communities among different niches of white rhinoceroses have effects on each other and build a complex linkage.

### Captive ground influences the bacterial communities of white rhinoceroses.

The white rhinoceroses in this study were raised on three different grounds (named 4_5, 6, and 12), and each ground has five white rhinoceroses. As the above results indicated that bacterial communities of the gut, skin, and environment have an interaction, we then explored how the captive ground influences the bacterial communities of the white rhinoceroses. By characterizing the beta diversity of the gut, skin, and environment samples, respectively, we found that the samples from different captive grounds were separated from each other (Fig. S5A to C). We found that the main composition and structure of the microbiome within the same niches were similar at the phylum level regardless of breeding grounds. As shown in Fig. S5D, the dominant phyla of bacteria in the gut microbiome of white rhinoceroses from the three grounds all were *Firmicutes*, *Bacteroidota*, and *Patescibacteria*, while the dominant phyla of bacteria in the skin and environment microbiome both included *Actinobacteriota*, *Chloroflexi*, *Proteobacteria*, *Acidobacteriota*, and *Myxococcota*. At the genus level, we found that *Oscillospiraceae* NK4A214 group, *Christensenellaceae* R-7 group, *Rikenellaceae* RC9 gut group, *Lachnospiraceae*, *Saccharofermentans*, *Anaerovorax*, and *Lachnospiraceae* AC2044 group were the most abundant genera in the gut of the white rhinoceroses in all the three grounds; in contrast, the abundant genera in the skin and environment data set from the three grounds were a little different, especially the percentage of JG30-KF-CM45, *Nocardioides*, *Arthrobacter*, and SBR1031 (Fig. S5E). The Venn diagram showed that 16 phyla and 41 genera were shared by all the samples, while the samples from the 4_5 ground has more unique species, and the environment samples from the ground 6 has the most unique genera compared to other groups (Fig. S5F and G). These results indicated that the captive ground influences the bacterial communities of white rhinoceroses.

### Diversity of the gut and skin bacterial community varies with age.

**FIG 5:**
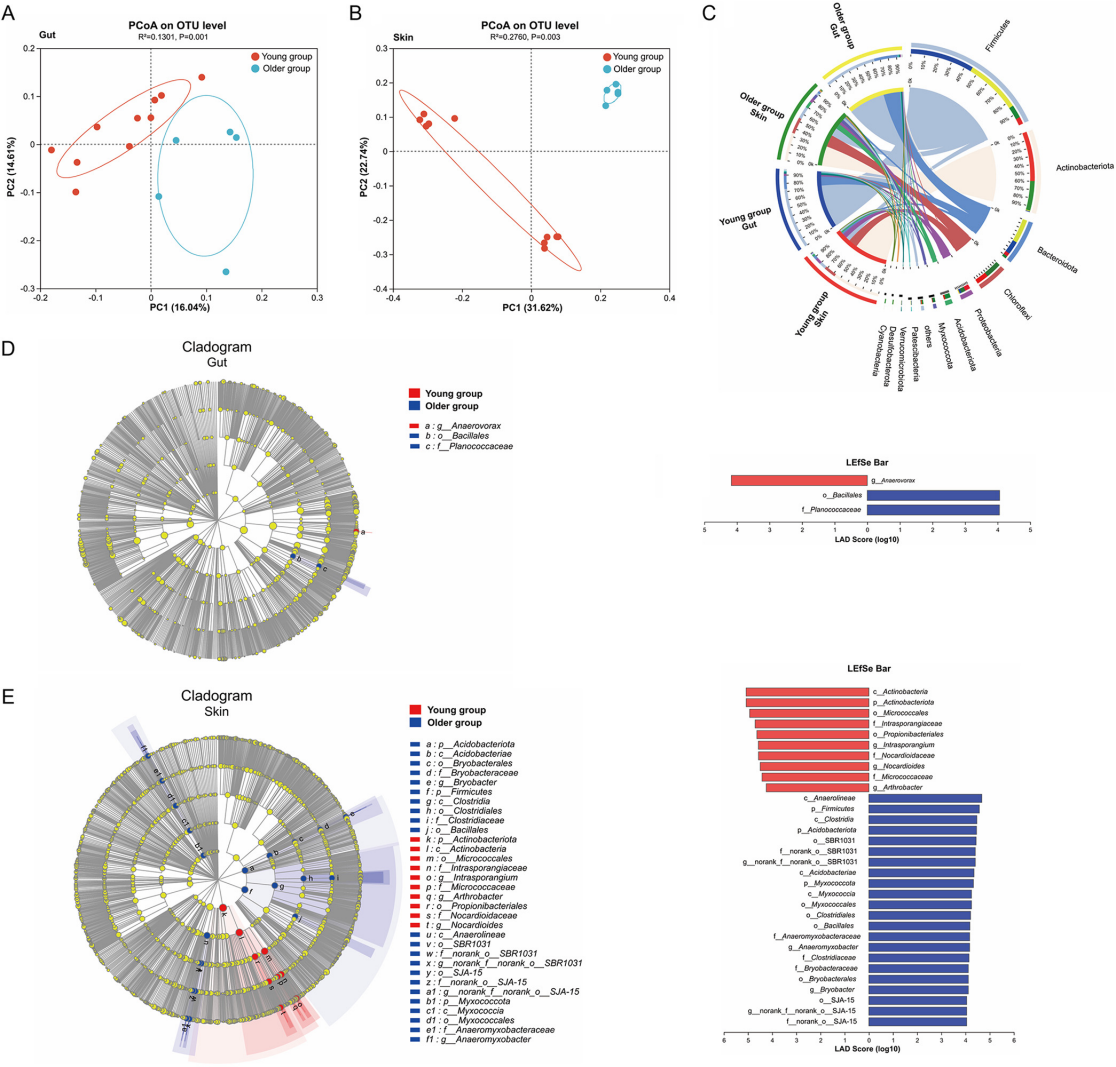
Differentially abundant microbial taxa between white rhinoceroses of different ages. Beta diversity of gut (A) and skin (B) microbiota of white rhinoceroses in different ages displayed in a PCoA scatterplot. The PCoA analysis was performed based on Unweight_UniFrac distance at the OTU level. (C) The relative abundances of the gut and skin bacterial community of white rhinoceroses in different ages at the phylum level. Phyla with relative abundance < 1% were defined as others. (D, E) The biomarkers in the young and older groups were identified by the LDA using LEfSe. The circle radiating inside-out in the cladogram indicates the classification (from phylum to genus). The diameter of each circle is proportional to the relative abundance of the bacterial species. The red and blue circles demonstrate the biomarkers by different groups, while the yellow circles demonstrate the species are nonsignificant. The right histogram displays the LDA score (>4) of the biomarkers with significant differences between the groups.

Previous studies showed that age is associated with modifications in the composition and dynamics of the gut bacterial microbiota (22, 23). Here, we analyzed how age influences the bacterial communities of white rhinoceroses. The adult white rhinoceroses were divided into two groups by age: the young group (between 5 and 8 years old) and the older group (between 13 and 16 years old) (Table S1). By characterizing the beta diversity of the gut and skin samples of the young and older white rhinoceroses, we found that the young and older samples were separated from each other (Fig. 5A and B). Although the predominant phyla of the gut and skin samples were similar, respectively, in the young and older white rhinoceroses, the percentage of the bacterial species was much different (Fig. 5C). For example, the predominant phyla of the gut microbiome of the older group were *Firmicutes* (65%) and *Bacteroidota* (29%); however, the young group has higher *Firmicutes* (75%) but lower *Bacteroidota* (16%) than the older group; similarly, the skin microbiome of the older group has more *Firmicutes* (15% versus 8.4%) and *Chloroflexi* (20% versus 15%), but less *Actinobacteriota* (35% versus 59%) than young group (Fig. 5C). The linear discriminant analysis effect size (LEfSe) with a linear discriminant analysis (LDA) value of > 4 was further applied to assess the specific taxonomic differences between the young and older groups of white rhinoceroses. We found that *Anaerovorax* at the genus level was more abundant in the gut microbiome of young groups, while *Bacillales* at the order level and *Planococcaceae* at the family level were more abundant in the older group (Fig. 5D). As for the skin microbiome, 32 bacterial taxa were identified as the differential flora between the young and older groups, which includes 4 phyla, 5 classes, 8 orders, 8 families, and 7 genera (Fig. 5E). These results indicated that age may have induced shifts in the microbial community composition of the gut and skin of the white rhinoceroses.

## DISCUSSION

Our planet is currently undergoing a sixth mass extinction, especially for the large mammal species, which is driven by human activities (24, 25). The white rhinoceros is one of the largest mammalians on the land. It needs a long period of time for sexual maturity and pregnancy, and thus the population was easily threatened by habitat destruction and poaching (3). The microbial population plays a key role in animal health and welfare (7, 19); however, studies regarding the microbial communities of the white rhinoceros are relatively limited. Here, we collected fecal, skin, and environment samples from 15 captive white rhinoceroses and attempted to analyze the microbial diversity and interaction of the bacterial communities from the three different niches.

As an ungulate animal with three toes on each hoof, the white rhinoceros is closely related to the horse and they have a similar digestive system (26). Previous studies showed that *Firmicutes* (68%) and *Bacteroidetes* (14%) predominated in the feces of healthy horses (27). Here, we found that the gut microbiome of white rhinoceroses was mainly occupied by the phyla *Firmicutes* (72.07%) and followed by *Bacteroidota* (20.26%), which suggested that the predominant phyla of the feces of the white rhinoceroses and healthy horses were similar. Both the white rhinoceroses and horses belong to the hindgut fermenter, and they could utilize fibrous feeds and gain energy through microbial fermentation (27, 28). Previous studies showed that the most abundant phylum in the hindgut of healthy mammals was *Firmicutes*, which is related to the fermentation of insoluble fiber in the host cecum and large colon (15, 29). In this study, the cooccurrence network further indicated that *Firmicutes* may play most roles in the hindgut fermentation of white rhinoceroses. Within the *Firmicutes* phylum, we found that the *Oscillospiraceae* NK4A214 group, *Christensenellaceae* R-7 group, and Eubacterium coprostanoligenes group were the most predominant genera in the feces of the white rhinoceroses. The *Oscillospiraceae* NK4A214 group and Eubacterium coprostanoligenes group belong to acetate- and butyrate-producing bacteria, respectively, which were associated with the development of metabolic disorder (30–32); the *Christensenellaceae* R-7 group was presumed to play a role in influencing intestinal morphology (33). Here, we found that *Bacteroidota* was the second most abundant in the gut microbiome of white rhinoceroses. This phylum is also predominated in the gut of other herbivores (34). Within the *Bacteroidota* phylum, the genus *Rikenellaceae* RC9 gut group is the most abundant in the feces of the white rhinoceroses. This genus has important roles in alleviating gut inflammation and breaking down plant-derived polysaccharides (35). These dominated bacterial species may determine the functional gene abundance in gut bacteria of the white rhinoceroses, such as carbohydrate metabolism, amino acid metabolism, and energy metabolism (Fig. 1C).

Captive breeding is crucial for the conservation of the white rhinoceros and contributes to the physiology study of the threatened species. In the captive ground, soil is an important part of animal habitat, providing food, water, and shelter (10). Soil microbiota is highly diverse, and could be absorbed by food, plants, water, and others (10, 11, 36). Previous study showed that the gut microbial diversity was increased when the BALB/c mice exposure to soil microbes (37), which suggested that soil biodiversity is interrelated with the gut microbiome. Here, we observed the interaction between the host (gut and skin) microbiome and the environment/soil microbiome of the captive white rhinoceroses. Additionally, we found that the skin and environment share similar microbiome profiles (Fig. 2). Skin is the outermost surface of the body, which like the host intestinal tract is interconnected with complex bacterial communities (19, 20). As the mud bath is a common behavior of white rhinoceroses, both the intestinal environment and skin are subject to a constant influx of soil microbes, and thus the skin microbiome of white rhinoceroses could be largely affected by the environment. Previous study reported that skin microbes participate in numerous physiological processes, especially the immunological processes, and thus indirectly affect the intestinal microbiome (19). Meanwhile, the gut microbiome also has a significant impact on skin health (21). Here, our results showed that a bacterial linkage was built by the gut, skin, and environment of the white rhinoceroses, which suggested that the outer environment could continuously interact with the host microbes and may influence the body health. The interrelation of the host microbiome and environment microbiome has also been assessed in previous studies. For example, the skin mucus communities of Teleost fish are highly environment-dependent (38), and the gut microbiota of infants was influenced by the household furry pets (39). Nonetheless, the major bacterial composition of the gut significantly differs from that of the skin and environment in white rhinoceroses (Fig. 3). These could be due to the different conditions among the niches, such as the oxygen, moisture, pH, and nutrient.

The number of studies that documented the effect of age on gut microbial communities has increased in recent years (22, 23). Previous study showed that the developing gut microbiome of humans from infancy to childhood undergoes three distinct phases of microbiome progression (40); the composition and function of the human gut microbiota change with aging, which in turn affects human health (22). A study of tigers also found that the composition of gut microbiome undergoes significant changes with age, which could be divided into three distinct phases (41). In this study, we found that the beta diversity of the gut and skin samples of the young and older white rhinoceroses both showed a significant difference, respectively. Further studies indicated that the microbiome profile of the gut and skin both have an obvious difference in the composition of microbial communities based on the ages of white rhinoceroses. For example, the proportions of *Firmicutes* were decreased and the proportion of *Bacteroidota* increased in the gut of the older group compared with that in the young group, while the proportions of *Firmicutes* and *Chloroflexi* were increased and the proportion of *Actinobacteriota* decreased in skin of the older group compared with that in the young group. As aging is always accompanied by a decline in immunity and gut barrier function, as well as various other disorders, it is easy to understand the age-related changes in the gut and skin microbiota. Additionally, our results also showed that the captive ground influences the bacterial communities of white rhinoceroses (Fig. S5). These findings further indicated that the bacterial linkage based on the interaction of the bacterial communities from different niches is dynamic.

In summary, our study describes the characteristics and interaction of bacterial communities of the white rhinoceros in three different niches, including gut, skin, and environment. We also analyzed the different effects of breeding ground and age on the composition of the bacterial community. These observations provide comprehensive information on the bacterial communities of this endangered species and may have important implications for the protection and management of the captive white rhinoceros. Whereas this study has many strengths, there are also some limitations. It is noticeable that v1–v3 variable region of the 16S rRNA gene had been suggested to be a better choice for skin microbiota in humans among others (42). Therefore, the imperfection is that the 16S region analyzed here may underestimate some taxa like Cutibacterium acnes in the skin. Another caveat to our analysis is that the accuracy of the PICRUSt2 functional predictions may be lower than in other studies. Future studies should use metagenomics and metabolomics to identify the effectors that affect the bacterial linkages between the white rhinoceros and its environment and explore their associations with animal health.

## MATERIALS AND METHODS

### Sample collection.

A total of 15 healthy adult white rhinoceroses were sampled between April and June in 2021. These white rhinoceroses were averagely raised on three different grounds named 4_5, 6, and 12, at the Breeding and Research Center of Wildlife (E: 103.39°, N:24.90°) in Shilin County, Yunnan Province, China. Every captive ground covers an area of some 30,000-square meters. The twice-daily diet for each white rhinoceros consisted of 16.5 kg hay pellets (7.5 kg *Leymus chinensis*, 7 kg alfalfa, and 2 kg oat grass), 0.5 kg carrot, 0.5 kg maize flour, 0.3 kg wheat bran, and 15 kg cornstalk or other fresh gramineous pasture, such as wheatgrass, *Pennisetum sinese*, and green manure. The captive ground, age, and sex of the white rhinoceroses are presented in Table S1. Fresh fecal samples were immediately collected from each animal upon defecation in the morning. Skin samples were collected from the head, neck, and back of the white rhinoceros using skin surface swabs. Soil from a 3 cm depth in the mud bath sites of the white rhinoceros was collected and used as environment samples. The environment samples were collected at different locations in the captive ground with a distance of about 1 m. Each captive ground has five environment samples. In total, we obtained 15 fecal samples, 15 skin samples, and 15 soil samples, which were all collected in sterile containers and stored at −80°C for further analysis.

### DNA extraction and 16S rRNA amplicon sequencing.

The total DNA was extracted from the samples using DNA kit according to the instructions of the manufacturer, which is designed for the rapid purification of high-quality genomic DNA from fresh or frozen samples. The fecal samples were processed by QIAamp Fast DNA Stool minikit (Qiagen Inc., Germany), while the skin and soil samples were processed by Qiagen Powersoil DNA extraction kit (Qiagen Inc., Germany). Three blank controls of PBS buffer were also processed identically to all samples for DNA extractions and sequencing. The quality and quantity of the DNA sample were determined by using a NanoDrop device (ThermoFisher Scientific, USA), and then the DNA samples were stored at −80°C for subsequent analysis.

Here, the v3-v4 variable region of the 16S rRNA gene was targeted via the specific primers (338F-806R), 338F: ACTCCTACGGGAGGCAGCAG, and 806R: GGACTACHVGGGTWTCTAAT. The purification, quantification, and homogenization of the PCR amplification products are performed by using the TruSeq DNA PCR-Free Sample Preparation kit (Illumina, USA). After that, the sequencing libraries were obtained for quality inspection, which is processed by the Agilent Bioanalyzer 2100 system (Agilent Technologies Inc., USA) and the Qubit@ 2.0 Fluorometer (ThermoFisher Scientific, USA). An Illumina NovaSeq PE250 platform (Illumina, San Diego, USA) supplied by Majorbio Bio-Pharm Technology Co. Ltd. (Shanghai, China) was used to sequence the library, and 250 bp paired-end reads were generated.

### Data analysis.

After the sequencing, the quality of the reads was assessed using FastQC (V0.11.8) (https://www.bioinformatics.babraham.ac.uk/projects/fastqc/). All reads were sorted, screened, and filtered by the Trimmomatic (V0.33), FLASH (V1.2.7), and QIIME2 (V2021.8) (43, 44), which help to ensure the quality and length. Sequences with ≥ 97% similarity were then assigned to the same operational taxonomic unit (OTU) by using Uparse software (V7.0.1001, http://www.drive5.com/uprase/) (45). The representative sequence for each OTU was screened for further annotation, which is processed by using the Silva Database (http://www.arb-silva.de/) based on Mothur algorithm to annotate taxonomic information (46). The abundances of OTU were normalized using a standardized sequence number, which was used for the subsequent analysis.

Beta diversity, displayed as principal coordinates analysis (PCoA), was calculated using QIIME2 based on the Bray–Curtis and UniFrac distances and plotted in R (47). PICRUSt2 software was used to predict the function of the microbial community at the KEGG pathway level2 (48). Ternary plot analysis was performed in R package ggplot2, DESeq2, and grid to investigate the enriched and depleted bacterial community among different groups. Venn diagrams (Venny 2.1.0) were applied to visualize the overlapping and unique enriched bacterial species among different groups. The network of cooccurrences of bacterial communities was constructed based on Spearman's correlation (|R| > 0.8, FDR-adjusted *P* < 0.05). Gephi software (V0.9.2) was applied to visualize and modularize microbial correlation networks. The Linear Discriminant Analysis (LDA) Effect Size (LEfSe) was performed to identify differentially abundant taxa between different groups, while an LDA score threshold of 4.0 (*P* < 0.05) was set. All these statistical analyses were performed using R (https://www.r-project.org/).

### Data availability.

The data sets presented in this study are available in the NCBI primary data archive (PDA) with accession number PRJNA935850 and can be found at https://www.ncbi.nlm.nih.gov/sra/PRJNA935850.
