# Supplementary material for: High-Throughput Sequencing Reveals a Dynamic Bacterial Linkage between the Captive White Rhinoceros and Its Environment
Source: Microbiol Spectr. 2023 Jul 6;11(4):e00921-23. doi: 10.1128/spectrum.00921-23 (PMC10434261; doi:10.1128/spectrum.00921-23)
Supplement: Supplemental material — Download spectrum.00921-23-s0001.pdf, PDF file, 0.8 MB [file spectrum.00921-23-s0001.pdf]

Supplemental material for:

High-throughput sequencing reveals a dynamic bacterial linkage between the captive white rhinoceros and its environment

Xiaojun Zhong[a#], Junyang Zhao[a#], Ying Chen[b#], Yanxin Liao[a], Tao Qin[c], Dingjiang Zhang[b], Xiaogang Lai[b], Chunlong Yang[b], Yu Wang[b]*, Xianfu Zhang[a]*, Menghua Yang[a]*

[a]College of Animal Science and Technology, College of Veterinary Medicine, Zhejiang A & F University, Key Laboratory of Applied Technology on Green-Eco-Healthy Animal Husbandry of Zhejiang Province, Zhejiang Provincial Engineering Laboratory for Animal Health Inspection & Internet Technology, Hangzhou, 311300, China

[b]Yunnan Shilin Longhui Wildlife Research Center Co., Ltd, Kunming, 652200, China

[c]State Key Laboratory of Subtropical Silviculture, College of Forestry and Biotechnology, Zhejiang A & F University, Hangzhou, 311300, China

[#] These authors contributed equally to this work.

[*] Corresponding author: Menghua Yang, Xianfu Zhang, and Yu Wang,

Supplemental Table S1 Information on the white rhinoceros used in this study

Rhinoceros	Age (years)	Captive ground	Sex
59	15	4_5	male
36	15.5	4_5	female
63	15.5	4_5	female
70	15.5	4_5	male
90	14.5	4_5	female
105	7	6	male
106	7.5	6	female
97	8	6	male
108	7	6	female
112	7	6	female
109	6	12	male
113	6	12	male
116	6	12	female
120	5	12	female
125	5	12	male

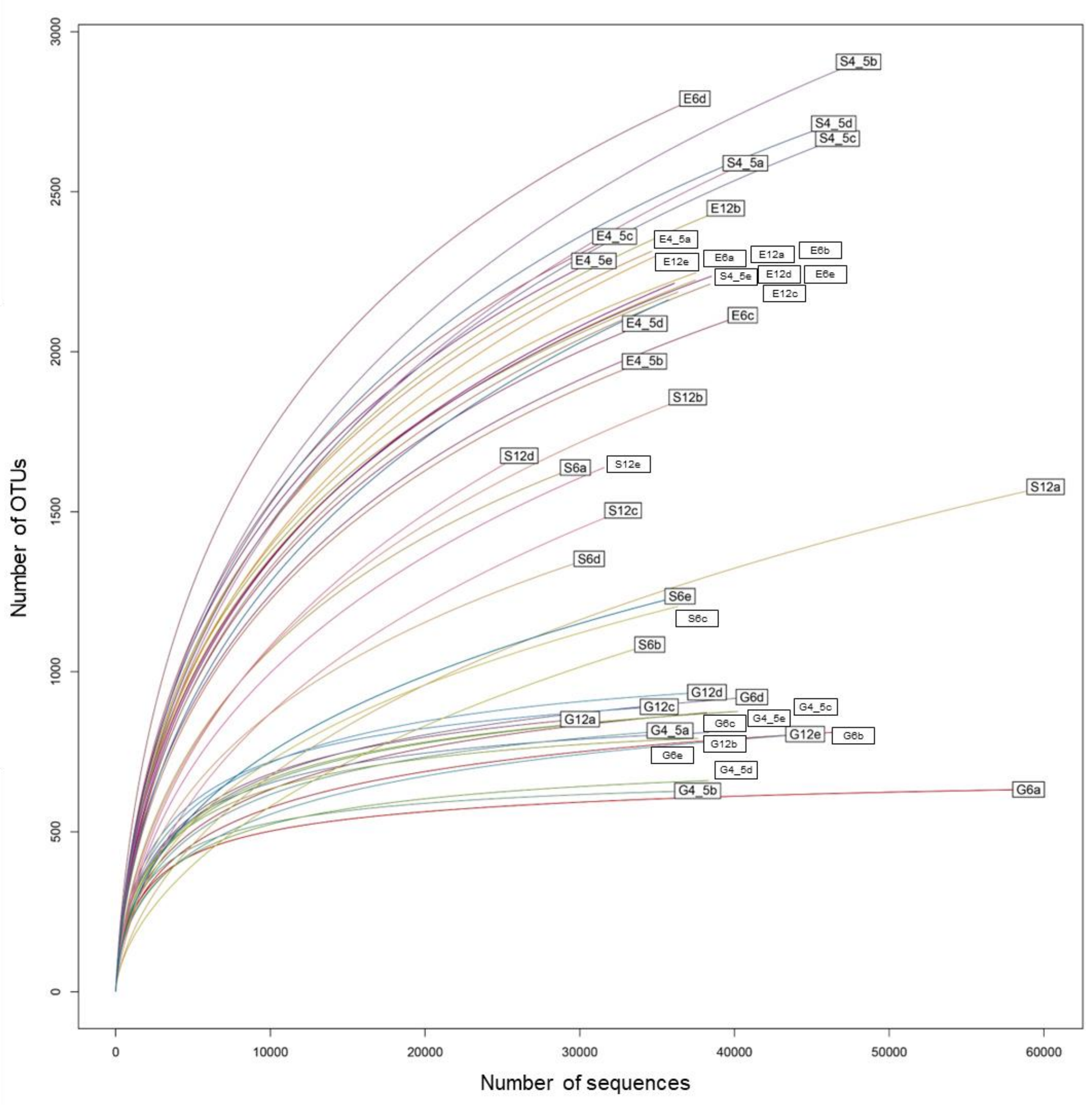

Supplemental Figure S1 Rarefaction curves. Rarefaction curves comparing the number of reads with the number of phylotypes found in the DNA in all samples.

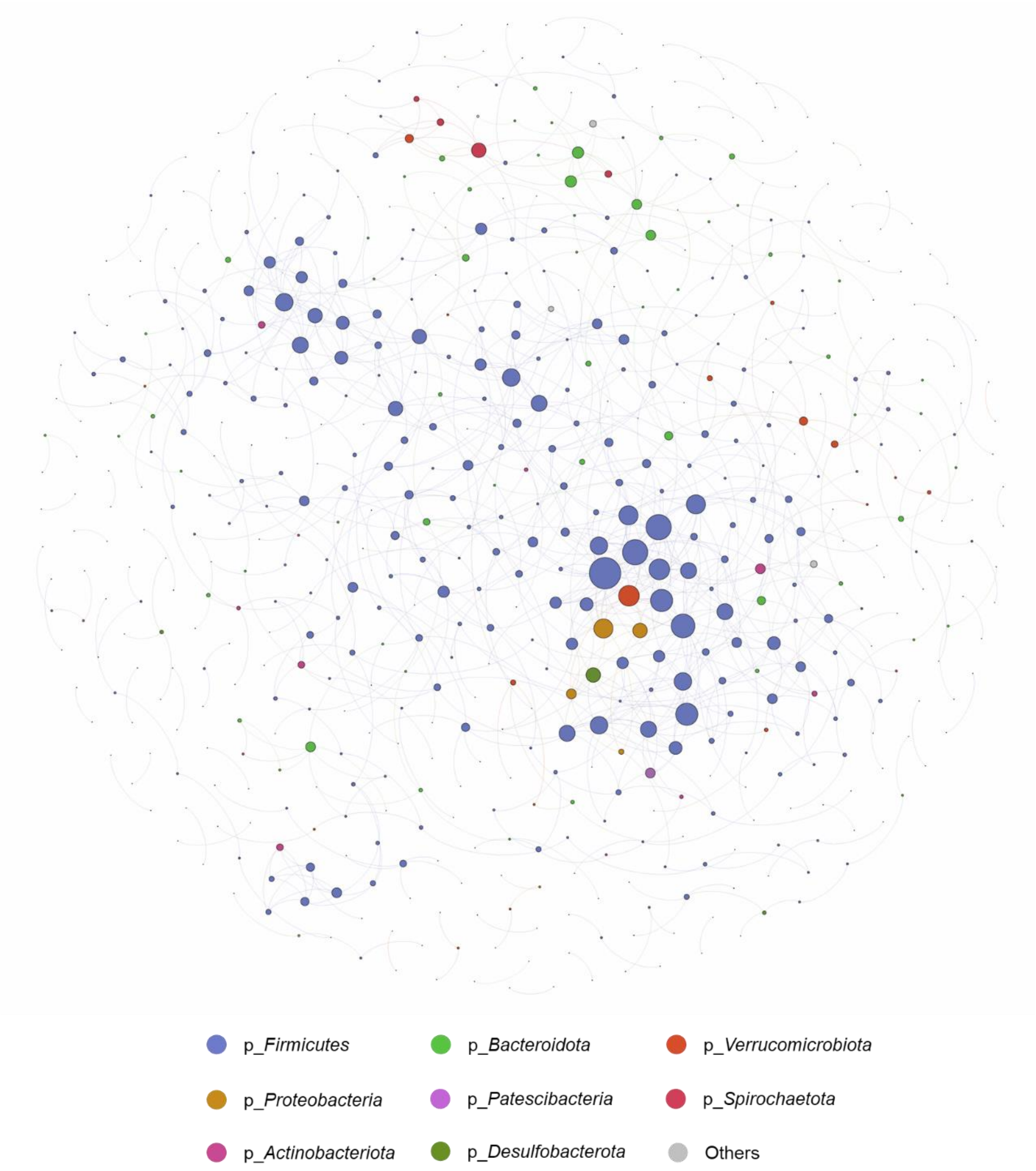

Supplemental Figure S2 Co-occurrence networks of the gut microbial communities. The connections in the network represent a strong (Spearman's R > 0.8) and significant (FDR-adjusted $p < 0.05$) correlation. The color of each node represents different major phyla (relative abundance > 0.5%), and the size is proportional to the relative abundance.

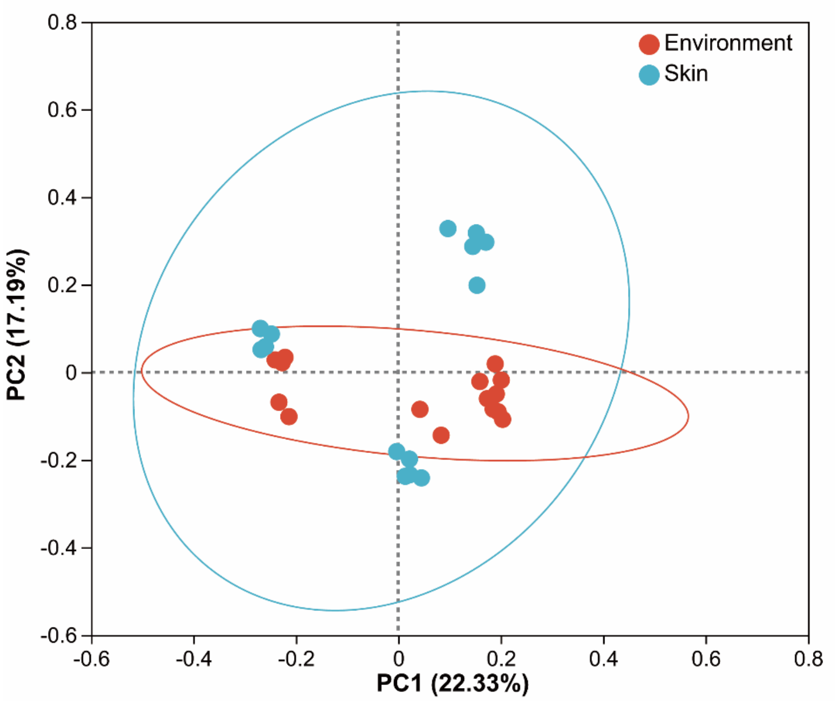

Supplemental Figure S3 Beta diversity shown in a principal co-ordinates analysis (PCoA) scatterplot. The PCoA analysis was performed based on Unweight_UniFrac distance at the operational taxonomic unit (OTU) level.

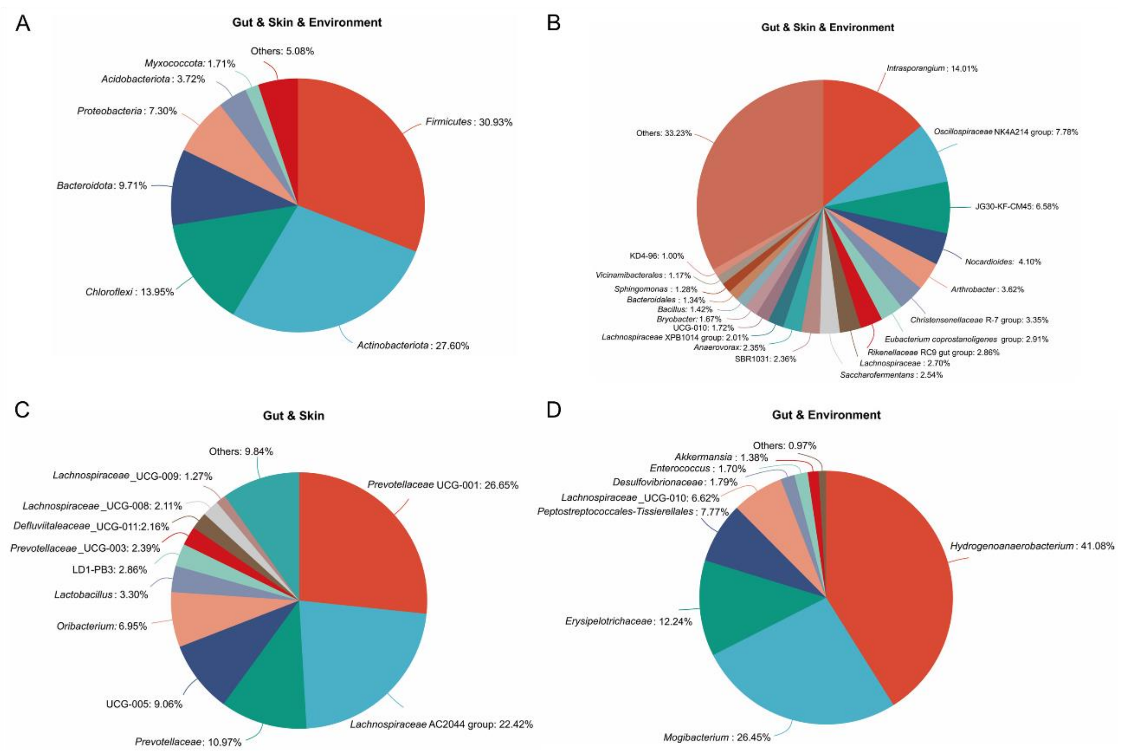

Supplemental Figure S4 The composition and proportion of bacterial communities shared by the different samples. (A) The composition and proportion of the phyla shared by the gut, skin, and environment groups. The parts with different colors represent the different phyla. **(B)** The composition and proportion of the genera shared by the gut, skin, and environment groups. **(C)** The composition and proportion of the genera shared by the gut and skin groups. **(D)** The composition and proportion of the genera shared by the gut and environment groups.

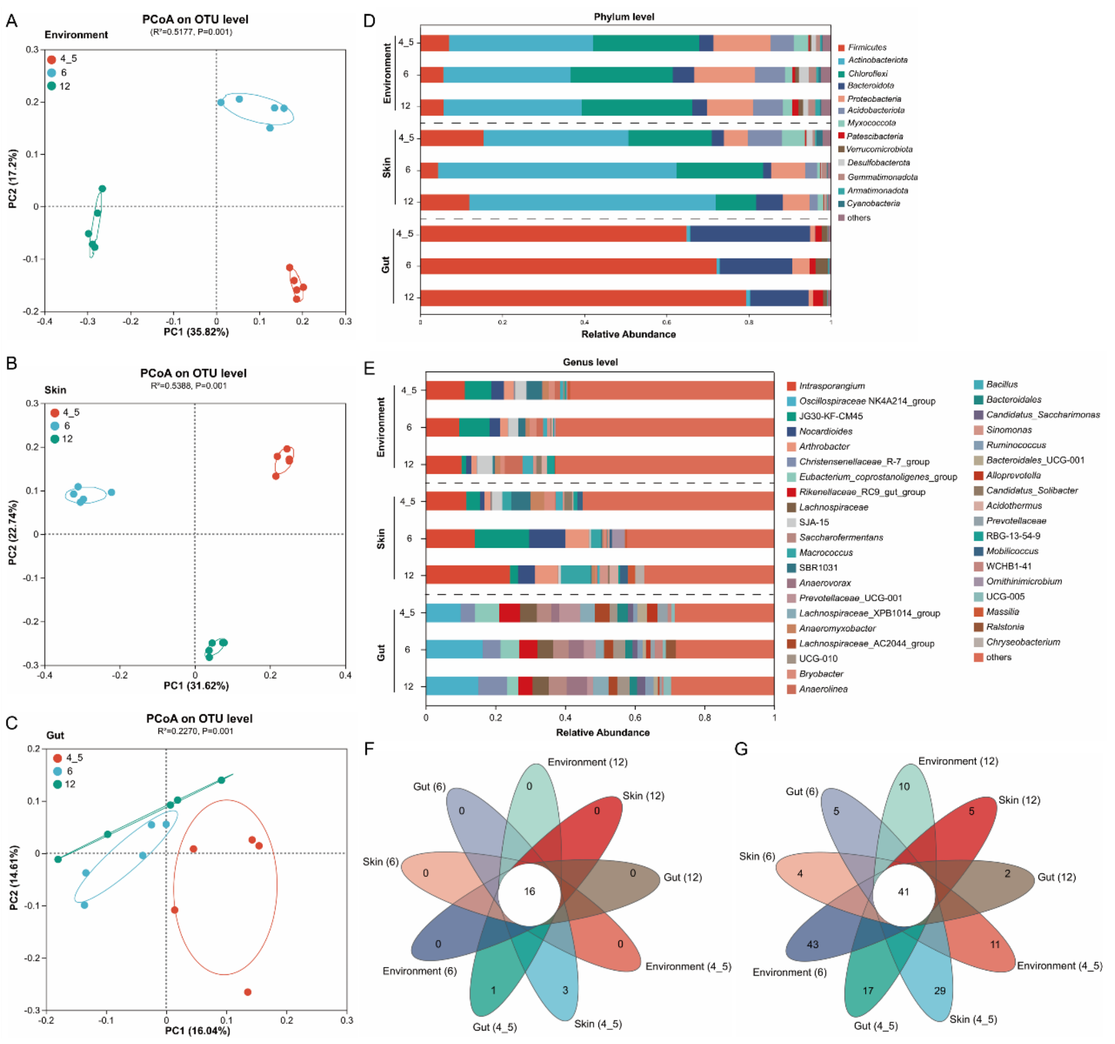

Supplemental Figure S5 Bacterial composition of the white rhinoceroses from different captive grounds. Beta diversity of environment **(A)**, skin **(B)**, and gut **(C)** microbiota in different captive grounds displayed in a PCoA scatterplot. The PCoA analysis was performed based on Unweight_UniFrac distance at the OTU level. **(D)** Relative abundance of dominant microorganisms at the phylum level. Phyla with relative abundance < 1% were defined as others. **(E)** Relative abundance of dominant microorganisms at the genus level. Genera with relative abundance < 2% were defined as others. The colors of each block represent different bacterial species, and the width of the block is proportional to the relative abundance. **(F)** Venn diagram based on the phylum level of bacterial communities in the gut, skin, and environment of white rhinoceroses from different captive grounds. **(G)** Venn diagram based on the genus level of bacterial communities in the gut, skin, and environment of the white rhinoceroses from different captive grounds.
